# Potential Impact of Kangaroo Mother Care Combined With Structured Physiotherapy on Neurodevelopmental Outcomes in a Low-Birth-Weight Infant: A Case Report

**DOI:** 10.7759/cureus.113231

**Published:** 2026-07-23

**Authors:** Dipti D Kadam, Mandar Malwade, Marzia A Bijle

**Affiliations:** 1 Department of Pediatric Neurosciences Physiotherapy, Krishna College of Physiotherapy, Krishna Vishwa Vidyapeeth (Deemed to be University), Karad, IND

**Keywords:** general movements assessment, kangaroo mother care, low birth weight, neonatal behavioral assessment scale, neurodevelopment, nicu, physiotherapy

## Abstract

Preterm and low-birth-weight (LBW) infants have a significant risk of neurodevelopmental deficits from an underdeveloped brain and a stressful neonatal intensive care unit (NICU) environment. This is a case report of a single LBW preterm male infant (34 weeks corrected gestational age, birth weight 1.82 kg) treated with a structured seven-day physiotherapy protocol, delivered during Kangaroo Mother Care (KMC) sessions in the NICU. The infant had a poor repertoire GM (general movement) and immature primitive reflexes at baseline. The individualized, developmentally appropriate physiotherapy protocol was developed and implemented for seven consecutive days, with the infant in skin-to-skin contact during KMC, and the protocol consisted of four phases: tactile containment, kinesthetic stimulation, postural alignment, and empowering the caregiver. Pre- and post-assessments were conducted using Prechtl's General Movements Assessment (GMA) and Neonatal Behavioral Assessment Scale (NBAS). The infant responded to the seven-day intervention by moving from a poor repertoire GM to an improved GM repertoire with increased variability and complexity within the writhing phase, and by improving in 10/13 NBAS reflex domains, although spontaneous neurological maturation may also have contributed to these changes. During the intervention period, no adverse events were noted. This case report demonstrates that a structured physiotherapy prescription can be integrated with KMC in one LBW infant and highlights the need for further investigation of its feasibility and potential benefit in larger controlled studies.

## Introduction

Preterm birth and low birth weight (LBW) continue to be among the great public health problems worldwide. UNICEF and the World Health Organization (WHO) estimate that in 2020, 19.8 million babies were born with LBW (14.7% of all live births) [1]. Preterm LBW infants are a highly vulnerable population and are at high risk for adverse neurodevelopmental outcomes due to immature neurological systems, medical comorbidities, and prolonged exposure to the stressful NICU environment [2]. Most preterm infants spend their time in the NICU, while the most rapid period of human brain development is between 22 and 40 weeks of gestation [3]. Interventions during this time of brain malleability can have a significant impact on later developmental outcomes. Despite advances in neonatal care, NICU environments induce excess sensory stimulation, painful procedures, and prolonged separation from caregivers, which are all linked to dysregulated responses of cortisol (the stress hormone) and a decrease in oxytocin (the bonding hormone), as well as altered brain maturation [4].

Kangaroo Mother Care (KMC) is defined as prolonged skin-to-skin contact (SSC) between the baby and a caregiver, with exclusive breastfeeding promotion and early discharge; it is recognized by the WHO as an essential intervention for all preterm and LBW babies [5]. KMC has been consistently linked to decreased neonatal mortality, improved thermoregulation, physiologic stability, and better mother-infant bonding. The results of a large meta-analysis indicated that KMC can reduce neonatal death by 36% in LBW infants [6]. Neurophysiologically, SSC stimulates C-tactile afferent fibers, leading to the release of oxytocin and endogenous opioids and inducing an autonomic regulation and a calming neuroregulatory state that facilitates sensory-motor integration [7].

Neonatal physiotherapy has become a key component of developmental care in NICUs, using positioning, passive range of motion, tactile stimulation, and sensory-motor activities to promote the development of the neuromotor system [8]. Structured tactile-kinesthetic stimulation in the neonatal period has been demonstrated to enhance motor organization, autonomic stability, and reflex maturation in preterm neonates [9]. Although there is an increasing number of evidence-based studies supporting KMC and neonatal physiotherapy as separate interventions, there are limited studies examining the implementation of a structured physiotherapy protocol within the KMC framework, especially when assessing the outcomes of the physiotherapy intervention in terms of neurobehavioral development as the primary outcomes [10].

A recent randomized controlled trial protocol by Reco and Soares-Marangoni [10] outlined a similar combined sensory-motor physiotherapy and KMC intervention in preterm infants of comparable postmenstrual age, using general movements as the primary outcome, though results from this trial are not yet available.

This is a case report of a seven-day structured physiotherapy program administered through KMC sessions and its outcome measured by Prechtl's General Movements Assessment (GMA) and Neonatal Behavioural Assessment Scale (NBAS) in one LBW preterm infant. The purpose of the report is to demonstrate the feasibility, safety, and potential benefit of this integrated approach and to help build the clinical evidence base for combined developmental interventions in the NICU. The parents of the infant gave written informed consent for the presentation of this case report and clinical data.

## Case presentation

Patient information

A preterm male infant, born at 34 weeks gestational age with a birth weight of 1.82 kg, was admitted to the NICU following delivery. The infant was the first child of a 26-year-old primigravida mother with an uneventful antenatal period. The infant was hemodynamically stable, did not require mechanical ventilation, and fulfilled criteria for KMC initiation. There was no family history of chromosomal abnormalities or neurological disorders. Parents' consent was obtained, and the infant was enrolled in the physiotherapy intervention program on Day 1 of admission to the NICU.

Clinical findings

Initial Assessment

Infant alert with stable vital signs. Neurobehavioral evaluation was done on Day 0 as a baseline. Poor repertoire general movement was detected with Prechtl's GMA during the writhing phase in spontaneous motor activity, showing suboptimal quality of spontaneous motor activity. The primitive reflex domains assessed by NBAS showed a baseline score of 1 (present/partial) for all reflex domains except for the domain of nystagmus, which was scored as 0 (absent), similar to normal at this gestational age. Lower limbs bilaterally are hypotonic for muscle tone. No prominent deformities, significant malformations, or active infections were detected. Muscle tone was assessed clinically by the primary investigator, under faculty supervision, through observation of resting limb posture and resistance to passive movement. A formal cranial nerve examination was not performed as a discrete component of this assessment; however, sucking, rooting, and glabella reflexes were evaluated as part of the NBAS primitive reflex battery. No other focal neurological deficits were noted on general observation.

Table 1 presents the chronological timeline of the infant's clinical course and the physiotherapy intervention period.

**Table 1:** Chronological Timeline of Clinical Events and Physiotherapy Intervention NICU: Neonatal Intensive Care Unit; KMC: Kangaroo Mother Care; GMA: General Movements Assessment; NBAS: Neonatal Behavioral Assessment Scale

Event	Date/Duration
Gestational age at birth	34 weeks
Birth weight	1.82 kg
NICU admission	Day 0
KMC initiation	Day 1 post-admission
Physiotherapy intervention started	Day 1 of KMC
Duration of physiotherapy intervention	7 days
Pre-intervention assessment (GMA and NBAS)	Day 0
Post-intervention assessment (GMA and NBAS)	Day 7

Diagnostic Assessment

Baseline NBAS assessment of primitive reflexes was conducted on Day 0. Thirteen reflex domains were assessed on a 0-2 ordinal scale (0 = absent; 1 = present/partial; 2 = fully elicited). The infant scored 1 in all domains except nystagmus (score: 0), which is developmentally expected at 34 weeks corrected gestational age. GMA video observation on Day 0 classified the infant's spontaneous movements as poor repertoire during the writhing phase, indicating reduced variation, speed, and amplitude in global movement patterns.

Table 2 presents the baseline and post-intervention NBAS reflex scores, demonstrating the pre-to-post change across all 13 domains.

**Table 2:** NBAS Primitive Reflex Scores—Pre- and Post-Intervention Score: 0 = absent; 1 = present/partial; 2 = fully elicited. NBAS: Neonatal Behavioral Assessment Scale; ATNR: Asymmetric Tonic Neck Reflex. ↑ indicates improvement from pre- to post-intervention.

Reflex Domain	Pre-Intervention (Day 0)	Post-Intervention (Day 7)	Change
Ankle Clonus	1	2	↑ +1
Sucking	1	2	↑ +1
Glabella	1	2	↑ +1
Plantar Reflex — Legs	1	2	↑ +1
Plantar Reflex — Arms	1	2	↑ +1
Placing	1	2	↑ +1
Standing	1	1	No change
Crawling	1	2	↑ +1
Incurvation	1	2	↑ +1
Tonic Head/Eye	1	2	↑ +1
Nystagmus	0	0	No change
ATNR	1	2	↑ +1
Moro	1	2	↑ +1

Intervention

Rehabilitation Course

A structured seven-day physiotherapy protocol was administered daily during KMC sessions by the primary investigator, under faculty supervision, as detailed below. All sessions were conducted while the infant remained in SSC with the mother. Continuous monitoring of infant stress cues, including color change, bradycardia, desaturation, and behavioral state, was performed throughout. Each session lasted approximately 20-30 minutes.

The intervention was administered by the primary investigator, a postgraduate trainee in Pediatric Neurosciences Physiotherapy, under the direct supervision of a senior faculty member holding a doctoral degree with extensive clinical experience in neonatal neurodevelopmental care. Session standardization was maintained by following the fixed seven-day, phase-wise protocol outlined in Table 3, with each day's session restricted to the designated focus area to ensure consistency in technique and sequence across sessions. Within each 20-30 minute session, time was allocated to infant state observation and settling, delivery of the day's primary therapeutic component, and continuous monitoring for stress cues, with sessions modified or shortened in response to any signs of infant distress. The protocol followed FITT principles: Frequency: once daily; Intensity: graded and cue-based; Time: 20-30 minutes per session; Type: progressive, goal-oriented physiotherapy. Table 3 outlines the week-wise, goal-oriented physiotherapy protocol implemented during the 7-day intervention period.

**Table 3:** Seven-Day Structured Physiotherapy Protocol Delivered During KMC KMC: Kangaroo Mother Care; PROM: Passive Range of Motion; NICU: Neonatal Intensive Care Unit

Day(s)	Focus Area	Therapeutic Interventions	Primary Goals
Days 1–2	Containment and Tactile Input	Hand placement on shoulders and hips; light touch; slow stroking of limbs	Promote flexed posture; activate C-tactile fibers for calming and sensory integration
Days 3–4	Kinesthetic Stimulation	Passive range of motion (PROM) for all extremities (5–10 repetitions); slow, graded joint movements	Maintain joint flexibility; provide kinesthetic input during skin-to-skin contact
Days 5–6	Postural Alignment	Prone-on-chest positioning; flexed supine positioning using towel rolls	Improve head control; promote body symmetry; counteract prolonged supine posturing
Day 7	Caregiver Empowerment	Education on feeding and sleep positioning; rocking techniques; tactile stimulation training for parents	Transition to family-centered care; ensure sustainable developmental support after NICU discharge

Results

After the seven-day physiotherapy treatment given during KMC, clinically meaningful improvement was observed in both neurobehavioral scores.

General Movements Assessment

Prechtl's GMA determined that the infant's spontaneous movements were ooor repertoire on Day 0 in the writhing phase. A post-assessment on Day 7 after seven days of physiotherapy showed improved quality of general movements, with greater variability, speed, and amplitude within the writhing repertoire, suggesting a clinically meaningful improvement in spontaneous motor activity. As the infant had not yet reached the postmenstrual age at which fidgety movements are expected to emerge (9-20 weeks post-term), true fidgety movements were not anticipated at this assessment; the observed change reflects a qualitative improvement within the writhing phase rather than a transition to the fidgety period. Table 4 summarizes the GMA outcome.

**Table 4:** General Movements Assessment (GMA) Pre- and Post-Intervention Outcomes Fidgety movements were not expected at this assessment given the infant's postmenstrual age; improved writhing movements reflect increased variability, speed, and amplitude compared to baseline. GMA: General Movements Assessment; PR: Poor Repertoire

Assessment Timepoint	GMA Classification	Outcome
Pre-intervention (Day 0)	Poor Repertoire (Writhing Phase)	—
Post-intervention (Day 7)	Improved Writhing Movements (increased variability/complexity)	Improved

Neonatal Behavioral Assessment Scale

The infant obtained a score of 1 on each of the 13 NBAS reflex domains at the baseline (Day 0) except nystagmus (score: 0). After the seven-day physiotherapy intervention, the infant's score was improved in 10/13 domains, to a score of 2 (fully elicited). There was no change in standing or nystagmus, as is expected at the current gestation. No deterioration was seen in any domain. Improvements in Moro, ankle clonus, sucking, plantar, placing, crawling, incurvation, tonic head/eye, glabella, and ATNR reflexes indicate better organization of neurobehaviors after physiotherapy and combined KMC. No bradycardia, desaturation, or color change was noted throughout the seven-day intervention period.

## Discussion

This case report illustrates the feasibility of a structured seven-day physiotherapy program given in the KMC setting and its positive neurodevelopmental outcomes in one LBW preterm baby. The infant improved in 10 of 13 NBAS primitive reflex domains and moved from poor repertoire GM to normal fidgety movements, and no adverse events were reported. The results were in line with and extended the growing evidence base supporting the embedding of neonatal physiotherapy into developmentally appropriate care systems. The secondary structure of C-tactile afferent fibers creates a neurophysiologically beneficial environment for KMC, providing oxytocin and endogenous opioid release and decreased pain sensitivity, which enables autonomic regulation [7]. During KMC, the infant is already in a parasympathetically dominant, hormonally regulated state, which is theorized to facilitate the integration of proprioceptive, vestibular, and kinesthetic inputs to a greater extent than either intervention could achieve alone [3,4]. It should be noted that these neurophysiological mechanisms were not directly measured in the present case and remain theoretical, based on findings from prior literature. The tactile containment and slow-stroking manipulation during Days 1-2 of the protocol are similar to the CT-activating stimuli described by Cascio et al. [11], which have demonstrated calming, neuroregulatory effects and support sensory-motor integration. Passive range of motion was introduced progressively on Days 3-4, which matched the physiotherapy approaches described by Sweeney et al. [12], which suggested cue-based, graduated sensory-motor stimulation based on individual infant readiness. The postural alignment activities on Days 5-6 specifically targeted a common issue of prolonged NICU stay: suboptimal positioning and limited active movement, which can lead to maladaptive posturing, and aimed to help the child achieve midline alignment and head control [8].

Structured tactile-kinesthetic stimulation has been shown to elicit profound changes in motor organization, weight gain, and autonomic stability in preterm infants by Field et al. [9]. Another systematic review by Alvarez et al. also found that the standardized use of massage therapy by trained personnel consistently enhances physiological stability and behavioral organization of hospitalized preterm neonates [13]. In this instance, the enhancement of NBAS reflex scores correlates with these findings and confirms the importance of using structured tactile-kinesthetic input to help facilitate reflex maturation. The change from poor repertoire to an improved, more variable writhing movement pattern seen at GMA is clinically relevant. True fidgety movements typically appear after 9-20 weeks post-term age, and their absence at the expected timepoint strongly predicts cerebral palsy and neurodevelopmental disorder [14]; however, as this infant's post-intervention assessment occurred well before that expected window, fidgety movements were not anticipated, and the observed improvement instead reflects a positive change within the writhing phase itself. While this improvement cannot be solely attributed to the physiotherapy intervention, due to the possibility of spontaneous maturation occurring over the seven-day period, the size and direction of change in GMA and NBAS indicate that the structured approach may have supported and/or accelerated the maturation of the brain system.

Day 7 focuses on the caregiver empowerment component of the family-centered model of care, offering parents opportunities to build the skills to carry on with developmentally supportive activities following their infant's NICU discharge. This is an important shift in order to maintain neurodevelopmental advances in the home environment and is congruent with goal-directed therapeutic approaches outlined by Lowing et al. [15]. It should be noted that due to the one-case design limitations, causal inference cannot be made. It is not possible to differentiate the contribution of KMC alone and physiotherapy alone, spontaneous neurological maturation, and the combination. Long-term follow-up data were not available to determine the longevity of the improvements observed. However, the findings from this case report represent a valuable clinical preliminary finding and template for protocol design for future controlled trials in this population.

Limitations and future directions

The present case report has limitations due to its single-case design, which limits generalizability and precludes causal inference. Due to the lack of a comparison group, it is not possible to determine the independent effects of structured physiotherapy, KMC alone, or spontaneous neurodevelopmental maturation on the outcomes measured. Long-term neurodevelopmental follow-up was not performed; thus, conclusions regarding the sustainability of improvements may be limited. The case report design did not allow for assessor blinding, thus potentially leading to assessment bias. Outcomes may also have been affected by variability in the NICU and the infant's medical condition. As larger study samples, controlled design, blinded assessment, and longer follow-up periods are recommended, future research is warranted in order to draw evidence-based conclusions regarding integrated physiotherapy-KMC treatment protocols for LBW preterm infants. Additionally, NBAS and GMA assessments were performed by a single, non-independently certified assessor under faculty supervision, without blinding to intervention status, which may have introduced assessment bias.

## Conclusions

This case report exemplifies the possibility and neurodevelopmental advantages of incorporating a structured seven-day physiotherapy program into KMC for a single LBW preterm infant, although causal inference cannot be drawn from a single case. The infant showed a clinically significant shift from poor repertoire to improved writhing movements on GMA and improved on 10 of the 13 NBAS reflex domains, with no adverse events observed during the intervention period. The findings suggest the feasibility and potential benefit of incorporating developmentally oriented physiotherapy into the already established neurophysiological framework of KMC for early neurobehavioral organization in this vulnerable group; however, conclusions regarding effectiveness cannot be drawn from a single case. The planned, progressive, and brief physiotherapy program, administered under appropriate supervision within KMC, appears safe and practically implementable in this instance. Larger controlled trials, with sample sizes employing similar combined intervention protocols, are required to further validate feasibility and potential benefit, optimize dosing parameters, and assess long-term outcomes.
